# Pinel: A Biographical Study

**Published:** 1860-09

**Authors:** 


ART. VII.—*Pinel: A Biographical Study.* By his Nephew, Dr. CASIMIR PINEL. Forbes's Journal of Psychological Medicine and Mental Pathology, April, 1860.

PINEL, the great medical philanthropist, was born at Saint-Paul-Capdejoux, France, April 20, 1745. Hoping that he would at some future day enter the church, his father bestowed upon him an excellent theological education, and was well repaid by the sincere piety and interest which his son manifested in his studies. At the age of twenty-two, however, Pinel renounced all idea of an ecclesiastical profession, and repaired to Toulouse, where he studied mathematics, and delivered lectures on philosophy. It was at this period that he wrote his thesis "On the Exactitude which the Study of Mathematics gives to the Judgment in its Application to the Sciences," which was very favorably received. A short time after he entered the Medical School at Toulouse, where, in 1773, after a brilliant examination, the degree of doctor of medicine was conferred upon him.

Longing for a greater field of knowledge, Pinel set out for the celebrated University of Montpellier, where, as at Toulouse, he soon attained the degree of doctor. As a means of support he entered a respectable family as tutor, and employed his leisure moments in composing theses for the aspirants for graduation.

In 1778, he left Montpellier for Paris, where he became a constant attendant at the hospitals, and wrote for several periodicals; among others the *Journal de Paris*, through whose medium he soon established a reputation as an able medical writer. In 1782, he assumed the editorship of the *Gazette de Santé*, in which appeared a portion of his treatise on Hygiene, and in 1784 translated, from the English, Cullen's "Institutions of Medicine." During the three following years he devoted much of his time to translations of works on chemistry, anatomy, surgery, materia medica, and pharmacy, and made many valuable literary contributions to the Academy of Sciences at Paris.

In 1783, owing to the loss of a friend who became a maniac on the subject of patriotism, Pinel devoted his whole attention to the study of mental aberration, and for five years pursued his observations in the *Maison de Santé Belhomme* with untiring ardor.

When not occupied in the manner just specified, much of his time was employed in writing articles relating to mental disorders, which appeared in the *Gazette de Santé*, still under his control. In 1791, he obtained the prize offered by the Royal Society of Medicine, for an essay "On the Most Efficacious Means of Treating Invalids who become Insane Before Old Age." The ability of this article was highly instrumental in securing to him the appointment which was conferred upon him the following year, of chief physician to the Hospital of Bicêtre. Then it was that he began that great and laudable reform which posterity should ever recognize with gratitude and veneration. It would be impossible for us, in our more enlightened age, to form even a faint conception of the wretched condition of the lunatic asylums at this period, and we cannot do better than to transcribe the description of them given by the author of this biography:—

"If we picture to ourselves low, damp, and infected dungeons, without light or air, containing a miserable stump-bed, or a rotten straw mattrass laid on the stone floor; if we imagine human beings naked, or covered with rags, nearly always furious, chained and shut up in these places of desolation and misery, real tombs, which they never left but for their last resting-place; if we conceive ferocious keepers, chosen from among convicts, treating these beings as brutes, making use of the most barbarous expedients, overwhelming them with injuries, mocking them with insulting jibes, mercilessly beating or waging with them terrible and often sanguinary struggles, throwing down before them disgusting and insufficient nourishment, leaving them without water to quench their thirst, or clothing to protect them from the cold of winter, and exposing them in this sad condition for the amusement of curious visitors; if we imagine these unhappy beings, believed to be incurable, abandoned by their families, deprived of medical care, pale, ghastly, and haggard, stagnating in their own defections, groaning under the weight of irons which lacerated their limbs, emaciated by repeated blood-letting, excited by their horrible sufferings and the inhuman treatment to which they were subjected, we shall then have a very incomplete idea of the frightful state of lunatic asylums in general, and of Bicêtre in particular, at this epoch."

In that revolutionary period, when a man's lightest action was canvassed and misinterpreted, Pinel was compelled to combat even more than ordinary difficulties and obstacles in the execution of his great enterprise.

The government was suspicious, and thought that in the request to grant some little freedom to lunatics there might be a latent plot, which would promote the interests of royalty and endanger those of the republic; so that it was not until after many struggles and oft-repeated petitions, that Pinel was permitted to liberate the insane from their fetters. This laudable action has been beautifully commemorated in a picture placed in the Academy of Medicine at Paris.

After an attendance of four years duration at the hospitals of Bicêtre and Salpétrière, Pinel published the results of his observations in his *Traité Medico-Philosophique sur l'Alienation Mentale.* At the same time appeared a work entitled *De la Philosophie de la Folie*, by Daquin, Pinel's contemporary. Daquin's friends claimed for him priority in the moral treatment of the insane, and pronounced his book to be infinitely superior to that of Pinel, a judgment as absurd as erroneous, for although it contains some statements worthy of notice and belief, they are so enveloped in a mass of worthless matter as to remind one forcibly of Virgil's mariners—"*Apparent rari nantes in gurgite vasto.*"

Pinel's work wrought a great reformation in the condition of lunatics. He introduced kind treatment, recommended moral remedies, and established a regular police in the asylums. He regarded physical treatment of minor importance, condemned bleeding, and strongly advocated delay. "What art cannot effect," he said, "time may accomplish."

In 1798 appeared Pinel's *Nosographie Philosophique*, which caused a complete revolution in the medical literature of the day, and for twenty years was the sole guide of physicians. This was his greatest work, the *ne plus ultra* of his ambition, and upon it he was well content to place his claims upon posterity, convinced that in the end his hopes would be fully realized.

In 1794, Pinel occupied the Chair of Hygiene, and afterwards that of Internal Pathology, in the *École de Santé.* He was nominated a member of the Institute in 1803, in the Zoological Section; Chevalier of the Legion of Honor; consulting physician to the Emperor, in 1805; Chevalier de St. Michael; honorary member of the Academy of Medicine; and was one of the eleven professors who witnessed the dissolution of the *Faculté de Médecine* under the ordinance of 1822.

Pinel was three times a competitor for the chair of *Docteur-régent de la Faculté*, and was each time defeated. His personal appearance was much against him; he was below the medium height; his manner was diffident, his voice weak, and his enunciation slow and wearisome; and we scarcely wonder when there were so many things to contend with externally, that an indiscriminating board should have bestowed the appointment upon a candidate of a more prepossessing exterior.

Pinel's modesty was so excessive that when he was presented by his friend Desfontaines to the king's aunts, with a view to being their physician, he stood before them perfectly dumb, and impressed the princesses so unfavorably that they at once declined his services.

Pinel was a friend of the unfortunate Marquis de Condorcet, and during the revolution of 1793 secreted him in the house of Madame Vernet, a relative of the renowned painter, Horace Vernet. This noble woman's residence afforded him an asylum for eight months; but learning from the papers that death was denounced against all those who concealed a proscribed person, he resisted her entreaties to remain, and left Paris in disguise. A few weeks after, he was found lifeless on the floor of his apartments, having taken poison, which he always carried with him.

Without being a very zealous Catholic, Pinel had a deep veneration for religion, to the truth of which assertion every action of his life bore tacit witness. One day he met the celebrated astronomer Lalande, who said to him: "I am preparing a new edition of the Dictionary of Atheists, in which I have devoted an article to you;" "and I," replied Pinel, "am about to publish my Treatise on Madness, in which I will reserve a place for you."

Pinel led a sober, moral life, and was endowed with an excellent constitution; he was rarely subject to sickness, but in 1793, while visiting the wards of Bicêtre, where typhoid fever was then very prevalent, he was attacked by this disease, which proved almost fatal to his life, and greatly impaired his general health. He often dwelt gratefully upon the fact that he chiefly owed his restoration to old Arbois wine, with which he ever afterwards kept his cellar well stored.

Pinel passed the greater part of his time at his country-house, between Etampes and Arpayon. Surrounded by an affectionate family, he resided here for more than twenty years, in comfortable though not affluent circumstances. In 1823, he was removed from the School of Medicine, on suspicion of entertaining liberal principles, and was left with an income totally insufficient to defray the expenses of his household. Thanks to the love and able management of his wife, Pinel was never cognizant of the straightened position to which he had been reduced; his habits remained unchanged and his every want was still readily and cheerfully supplied, though, in order to do this, Madame Pinel was often compelled to deprive herself of the barest necessaries of life.

In 1820, Pinel was seized with apoplexy, followed by partial paralysis, which recurred at unequal intervals during a period of five years. A great many persons assert that, in consequence of these attacks, his latter years were passed in imbecility. This is simply an error; for, though owing to the paralytic character of his disease, he was often incapable of expressing his ideas with freedom and rapidity, his intellect remained unclouded until the day of his death. The unerring diagnoses which he frequently made during the last year of his life are a sufficient corroboration of this statement.

On the 21st of October, 1826, he was attacked with pneumonia, which terminated fatally in three days. His remains were followed to Pére la Chaise by an immense crowd, composed of his friends and old pupils, who seemed greatly overcome by his death. In the procession were noticed many old women from the Salpétrière, who came to take a last farewell of him who, for more than thirty years, had been their best friend and benefactor.

Great and irreparable, indeed, was the loss which France sustained in his death. Talented, charitable, and humane, he was eminently capacitated for the work which he undertook and so thoroughly executed. Pinel can never be forgotten. Even after the lapse of centuries, when the mutability which rests upon the face of all things shall arbitrarily ignore names worthy of remembrance, and envelop in oblivion actions which now shed their lustre upon the world, the beneficent deeds of this great French psycopathist shall still remain fresh in the hearts of afflicted humanity, and shall place him among

> "The few and immortal names
> That were not born to die."

